# The Associations Among Self-Compassion, Self-Esteem, Self-Criticism, and Concern Over Mistakes in Response to Biomechanical Feedback in Athletes

**DOI:** 10.3389/fspor.2022.868576

**Published:** 2022-04-19

**Authors:** Yasamin Alipour Ataabadi, Danielle L. Cormier, Kent C. Kowalski, Alison R. Oates, Leah J. Ferguson, Joel L. Lanovaz

**Affiliations:** College of Kinesiology, University of Saskatchewan, Saskatoon, SK, Canada

**Keywords:** sport psychology, self-compassion, self-criticism, self-esteem, concern over mistakes, biomechanical feedback, sprinting, athletes

## Abstract

Athletes regularly face the possibility of failing to meet expectations in training and competition, and it is essential that they are equipped with strategies to facilitate coping after receiving performance feedback. Self-compassion is a potential resource to help athletes manage the various setbacks that arise in sport over and above other psychological resources. The primary purpose of this research was to explore how athletes respond to objective biomechanical feedback given after a performance. Specifically, we investigated if levels of self-compassion, self-esteem, self-criticism, and concern over mistakes were related to one another before and after a series of sprint tests interspersed with biomechanical feedback, and whether self-compassionate athletes achieved a better sprint performance after receiving and implementing biomechanical feedback. Forty-eight athletes (20 female: *M*_age_ = 19.8 years, *SD* = 3.1; 28 male: *M*_age_ = 23.6 years, *SD* = 7.8) completed online measures of self-compassion, self-esteem, self-criticism and concern over mistakes before performing four sets of 40-m sprints. Participants received personalized biomechanical feedback after each sprint that compared their performance to gold standard results. Following all sprints, they then completed measures of self-criticism, and reported emotions, thoughts, and reactions. Self-compassion was positively correlated with self-esteem (*r* = 0.57, *p* < 0.01) and negatively related to both self-criticism (*r* = −0.52, *p* < 0.01) and concern over mistakes (*r* = −0.69, *p* < 0.01). We also found that athletes with higher levels of self-compassion prior to sprint performance experienced less self-critical thoughts following biomechanical feedback and subsequent sprint trials (*r* = −0.38, *p* < 0.01). Although the results of this study provide some support for the effectiveness of self-compassion in promoting healthy emotions, thoughts, and reactions in response to sprint performance-based biomechanical feedback, a moderated regression analysis between the first and fourth sprint time variables revealed that self-compassion was not a moderator for change in sprint performance (*R*^2^ = 0.64, Δ*R*^2^ = 0.10, *p* > 0.05). These findings suggest that there are likely longer-term benefits of athletes using self-compassion to cope with biomechanical feedback, but that any benefits might be limited in a short series of sprint trials.

## Introduction

The interplay between an athlete's body and mind can have a significant impact on overall sports performance, particularly when athletes are expected to efficiently absorb and implement feedback within the competitive environment. Previous evidence suggests that feedback can significantly improve sports performance (Baudry et al., 2006; Mauger et al., 2009). While feedback can create desirable outcomes in sports performance, coping with feedback and effectively executing biomechanical improvement during competition and training can be challenging for some athletes (Mononen et al., 2003). As athletes regularly face the possibility of failing to meet expectations in training and competition (Gustafsson et al., 2017), it is essential that they are equipped with the dispositions and strategies that facilitate coping after receiving performance feedback. Although researchers have extensively examined the significant role certain psychological characteristics (e.g., resilience, self-belief, optimism, etc.) play in the development of sports expertise (Gould et al., 2002; MacNamara et al., 2010; MacNamara and Collins, 2015), little is known about how these psychological characteristics might facilitate an athlete's response to feedback given about their sport performance.

Biomechanical feedback provides objective technical information with the purpose of enhancing performance (Harfield et al., 2014), and it can be given about a variety of performance outcomes using several types of modalities. The feedback that is specifically related to performance results is known as *outcome feedback* (Salmoni et al., 1984). Outcome feedback can either be intrinsic or augmented—*intrinsic feedback* is the information provided by an athlete's own sensory and perceptual systems (McGill, 2001), while *augmented feedback* provides information that athletes do not receive from their sensory systems and is provided by an external source (Utley and Astill, 2008). Augmented feedback is often provided to athletes by coaches or trainers (Schmidt and Wrisberg, 2008). For example, coaches can give verbal information to athletes based on their observations in practice or comment on a sprinter's running technique from objective measurements (e.g., timing gates, video analysis, movement sensors, etc.). Augmented feedback can be provided using a variety of modalities—visual (e.g., screen), auditory (e.g., speaker), haptic (e.g., vibrotactile actuator), or a combination of the above (Akamatsu et al., 1995).

Augmented feedback can be further classified into *knowledge of result* (KR) and *knowledge of performance* (KP). KR is feedback about goal achievement (e.g., the time it takes to run a certain distance), while KP feedback is directed toward movement characteristics that influence performance outcomes (e.g., a runner's step length or frequency; Mononen et al., 2003). KR feedback is usually provided for tasks that require scaling of a single degree of freedom movement or a single dimension response (Salmoni et al., 1984). KP feedback usually provides information about the kinematics or kinetics of a movement (Newell and Carlton, 1987). The type of feedback an individual receives can shift the attentional focus of athletes. For example, receiving feedback about movement effects or outcomes (i.e., KR feedback) could result in an external focus, while feedback provided on body movements and movement coordination (i.e., KP feedback) might direct athletes' attention internally (Wulf, 2007). Due to the complexity of certain sports skills, KR feedback might not be the most effective type of feedback to provide to athletes, as it may prevent individuals from using intrinsic feedback processing and error detection (Salmoni et al., 1984). Consequently, athletes who are given KP feedback that addresses the kinematics of sports skills are more likely to experience successful sport outcomes (Schmidt and Lee, 1999).

One challenge faced by sport researchers, coaches, and trainers alike is that athletes may receive and implement feedback differently. More specifically, some athletes might perceive feedback negatively and experience a setback. The ability to overcome such a setback is essential for athlete success. Therefore, it is likely of great interest to various stakeholders (e.g., coaches, athletic directors, sport organizations, etc.) that athletes are equipped with the dispositions, skills, and resources to persevere when setbacks do occur, which would ultimately enhance athlete sporting experience, performance, and overall well-being.

Self-esteem has been acknowledged as a resource for athletes experiencing setbacks (Neff and Vonk, 2009; Mosewich et al., 2011). Self-esteem is “an evaluation of our worthiness as individuals, a judgment that we are good, valuable people” (Neff, 2011: p.1), and has been established as a pathway to fostering positive sport experiences (Boyer, 2007) and overcome negative challenges (Neff and Vonk, 2009). Despite the benefits of self-esteem in promoting positive self-evaluations (Boyer, 2007), happiness (Baumeister and Vohs, 2018), and self-confidence (Tilindiene et al., 2014) in athletes, relying solely on self-esteem might not be ideal (Mosewich et al., 2011; Neff, 2011). High levels of self-esteem are predicated on the “better-than-average effect” in that unrealistic positive self-evaluations are created through the process of putting down others to boost yourself up (Neff, 2011). Furthermore, enhancing self-esteem is difficult as it is resistant to change (Swann, 1996), and attempts to increase self-esteem are often unsuccessful (Neff, 2003a).

Self-compassion is understood as being kind and understanding toward oneself when faced with personal shortcomings and weaknesses (Neff, 2003a), and has been suggested as a potential alternative to self-esteem in helping athletes cope with some of the difficult challenges they might endure in sport (Mosewich et al., 2013; Ferguson et al., 2014; Sutherland et al., 2014). Though self-compassion and self-esteem are complementary concepts that are significantly and positively correlated with one another (e.g., *r*s = 0.56–0.59: Neff, 2003a; Leary et al., 2007), self-compassion has been shown to predict unique variance in well-being beyond self-esteem among athletes (Mosewich et al., 2011). Furthermore, self-compassion is positively associated with autonomy, environmental mastery, self-acceptance (Ferguson et al., 2021), and well-being (Ferguson et al., 2014), and has been significantly correlated with increased perceptions of athletic performance (Killham et al., 2018). The relationship between athletic performance and self-compassion might be due to highly self-compassionate athletes' decreased levels of self-criticism and fear of failure (Mosewich et al., 2011, 2013).

Self-compassion may be a valuable resource to attenuate setback experiences, such as receiving feedback that may be perceived as performance mistakes. For example, evidence suggests that self-compassion interventions led to decreased self-critical thoughts and concern over mistakes (Jopling, 2000; Gilbert and Procter, 2006). Leary et al. (2007) measured undergraduate students' responses to emotionally difficult situations encountered in their daily lives and concluded that self-compassion explained unique variance beyond self-esteem when predicting an individual's adaptive emotions, thoughts, and reactions to negative or emotionally difficult scenarios. Self-compassion can also play an important role in emotional distress regulation relative to a sports failure (Ceccarelli et al., 2019). Reis et al. (2015) asked 101 women athletes to respond to hypothetical and recalled sport events to examine the effect self-compassion might have during emotionally difficult sport situations. Results indicated that the emotions, thoughts, and reactions of highly self-compassionate women athletes were significantly more adaptive than their less self-compassionate peers. Mosewich et al. (2013) also investigated the effects of a self-compassion intervention on self-criticism and concern over mistakes in highly self-critical women athletes. Findings indicated that a 7-day psychoeducational and applied practice intervention effectively decreased self-criticism, rumination, and concern over mistakes. Moreover, the self-compassion levels of women athletes had significantly increased when measured post-test and during a one-month follow-up (η^2^ = 0.43). With these results in mind, it seems reasonable to speculate that self-compassion might be a relevant resource to help athletes deal with biomechanical feedback, particularly when the feedback presents an emotional challenge.

### Study Purposes and Objectives

While both adaptive psychological characteristics and the delivery of feedback can meaningfully contribute to athletic performance, it is important to examine how specific psychological characteristics might influence an athlete's response to feedback during sport-specific skills performance. The current study attempts to bridge sports biomechanics and sports psychology. More specifically, this study aims to explore how athletes emotionally respond to objective biomechanical feedback, and how self-compassion, self-esteem, self-criticism, and concern over mistakes are associated with those responses. A secondary purpose is to explore whether self-compassion can promote adaptive emotions, thoughts, and reactions in response to biomechanical feedback in athletes. As our intention was not to complete an exhaustive evaluation of all the psychological characteristics that can contribute to changes in athletes' responses to biomechanical feedback, we do not claim that the psychological characteristics we chose fully encompass an athlete's experience. However, self-compassion, self-esteem, self-criticism, and concern over mistakes have each been shown to be related to self-evaluation, and thus were best suited to evaluate our study hypotheses.

This study consisted of three groups of hypotheses based on time point and analysis type. Our first group of hypotheses were focused on the relationships between studied psychological variables at baseline. We hypothesized that (1a) pre-trials self-compassion would be negatively associated with baseline self-criticism and concern over mistakes in athletes, and that (1b) pre-trials self-compassion would be positively associated with self-esteem. Hypothesis two addressed the relationships between studied psychological variables before and after sprint testing. We hypothesized that (2a) pre-trials self-compassion and self-esteem would be negatively associated with post-trials self-criticism, and that (2b) pre-trials self-compassion and self-esteem would be positively associated with adaptive post-trials emotions, thoughts, and reactions. We also hypothesized that (2c) pre-trials self-criticism and concern over mistakes would be positively associated with post-trials self-criticism, and that (2d) pre-trials self-criticism and concern over mistakes would be negatively associated with adaptive post-trials emotions, thoughts, and reactions. Our third and final hypothesis was that (3) athletes with higher levels of self-compassion would experience improved sprint performances over baseline after receiving and implementing biomechanical feedback.

## Methods

### Methods Overview

This research was an interventional study with a within-between design where data was collected at multiple time-points. Approval to conduct the study was obtained from the institutional Human Research Ethics Board. Participants were recruited via various techniques including sending recruitment letters and posters to sport associations, coaches, and university athletes. Participation was voluntary, and all participants provided written, informed consent indicating that they fully understood the process and purpose of the study. All participants were treated in accordance with the ethical guidelines for human research set forth by the American Psychological Association. The study consisted of two phases. In Phase I, the biomechanical data of nine competitive University-level sprinters (five males, four females) were collected using timing gates and inertial sensors during three video-taped 40-m sprint tests. Sprinting was determined to be the ideal sport skill to examine our research objectives, as running is a fundamental form of human movement and is often used to evaluate athletic performance in elite and non-elite athletes (Lorenz et al., 2013). The most common measurements used to analyze sprinting performance are total sprint time and spatiotemporal stride characteristics (Bezodis et al., 2019), as stride length and stride frequency both play a key role in achieving maximum velocity in sprinting (Paruzel-Dyja et al., 2006). In addition to spatiotemporal parameters, trunk movement can also contribute to sprint performance (Kugler and Janshen, 2010). Phase I participant biomechanical information was used to establish gold standards for time, step length, step frequency, side-to-side (lateral sway), and front-to-back (anteroposterior sway) trunk movements. These gold standards were used as a reference for the biomechanical feedback delivered to participants in Phase II (instead of their own reference standard) with the goal to simulate prescriptive feedback (i.e., how to improve) and replicate learning environments where a coach gives feedback to help an early learner improve. While elite athletes can usually detect and correct their own errors in their primary sports, they might benefit from prescriptive feedback when learning new skills (Magill and Anderson, 2021).

**Figure 1:**
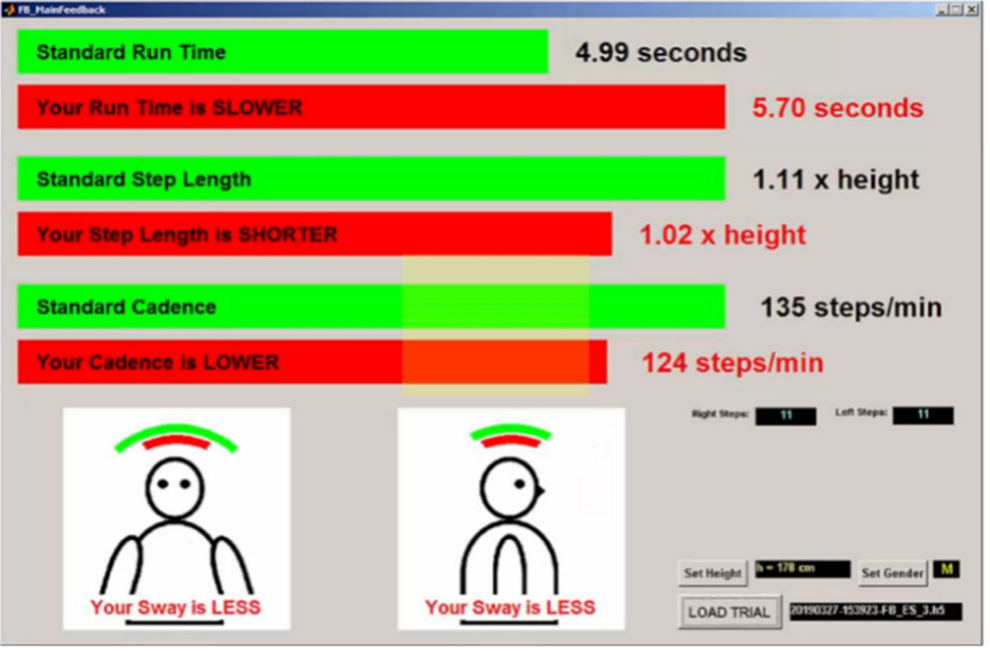
Sample biomechanical feedback presented to Phase II participants. Green bars indicate the sex-matched gold standard data. Red bars indicate the participant's performance for that trial. The rows display participant run time, step length, and cadence (i.e., step frequency). Step length is normalized to the participant's height. For trunk sway, the green arcs show the side-to-side and front-to-back gold standard trunk sways. The red arcs show the participant's sway.

Phase II involved collecting data from the main study participants. It consisted of one 60-min data collection session for each participant. At the start of the session, participant self-compassion, self-esteem, self-criticism, and concern over mistakes was measured via online questionnaires completed at the test site. Participants then performed four sets of sprint tests with timing gates and inertial sensors. The number of sets was chosen to minimize fatigue effects. Participants received biomechanical feedback after each sprint. The feedback contained a visual representation of their trial data in comparison to the gold standards established in Phase I. Participants were asked to improve their next sprinting performance using the biomechanical feedback that was provided (Figure 1). After performing all four sprint tests, participants completed a second online self-criticism questionnaire, as well as scales to rate how they reacted, felt, and thought in response to the biomechanical feedback (i.e., Performed Scenario Scale).

### Phase I (Gold Standard) Methods

#### Phase I Participants

To establish gold standards for the Phase II participant feedback, nine competitive collegiate-level sprinters (4 females: *M*_age_ = 22.0 years, *SD* = 3.3; and 5 males: *M*_age_ = 20.4 years, *SD* = 2.1) participated in this study. Separate gold standards were created for males and females.

#### Phase I Procedures

Data were collected on an outdoor varsity-standard running track. After a warm-up length of their preference, each sprinter wore three inertial sensors on their trunk and ankles (see Phase II Instruments section) and performed three sets of 40-m sprints on a straight path at maximum speed. Participants were given three minutes of rest time between trials. The collegiate-level sprinters did not receive any biomechanical feedback about the trials until after all data had been collected. The data from each participant's fastest trial were used to calculate the gold standard data for Phase II. Separate means for males and females were each calculated for sprint time, stride length, stride frequency, side-to-side sway, and front-to-back sway.

#### Phase I Results

See Table 1 for Phase I participant gold standard results.

**Table 1:** Descriptive statistics of biomechanical variables for Phase I and II participants.

**Variable**	***M***	**SD**
**Time (seconds)**
1st sprint	6.67	0.73
2nd sprint	6.40	0.54
3rd sprint	6.36	0.53
4th sprint	6.32	0.54
4th−1st sprint	0.40	0.40
Gold standard male	4.99	0.09
Gold standard female	5.57	0.28
**Step length (participant height)**
1st sprint	0.90	0.06
2nd sprint	0.95	0.05
3rd sprint	0.95	0.04
4th sprint	0.95	0.06
Gold standard male	1.11	0.14
Gold standard female	1.03	0.05
**Step frequency (steps/minute)**
1st sprint	120.3	9.4
2nd sprint	118.5	8.4
3rd sprint	118.7	8.1
4th sprint	119.9	8.3
Gold standard male	135.0	5.1
Gold standard female	129.2	3.3
**Front-to-back sway (degrees)**
1st sprint	20.3	5.3
2nd sprint	21.7	5.0
3rd sprint	21.9	5.1
4th sprint	21.6	4.6
Gold standard male	40.9	6.5
Gold standard female	48.5	6.9
**Side-to-side sway (degrees)**
1st sprint	9.3	3.0
2nd sprint	10.3	2.8
3rd sprint	10.9	3.4
4th sprint	11.2	3.4
Gold standard male	25.0	1.9
Gold standard female	27.1	3.7

### Phase II (Main Participants) Methods

#### Phase II Participants

Fifty athletes (22 females: *M*_age_ = 19.8 years, *SD* = 3.1; and 28 males: *M*_age_ = 23.6 years, *SD* = 7.8) participated in this study as main participants. Participants were excluded if they fell outside the age criteria (i.e., 16–35 years old). Participants were also excluded if they had not trained at least once a week within the past year for an organized sport. Two female participants were excluded from the study—one did not meet the age inclusion criteria and the other had incomplete sprint time data. Included athletes were involved with a variety of team sports including basketball (*n* = 9), football (*n* = 2), hockey (*n* = 2), soccer (*n* = 25), and volleyball (*n* = 10). Their current level of competition ranged from recreational (i.e., competing in intramurals or in a recreational league; *n* = 15), local (i.e., competing against athletes from your city/town; *n* = 13), provincial (i.e., competing against athletes from around your province; *n* = 8), regional (i.e., competing against athletes from other provinces; *n* = 8), national (i.e., competing at national championships; *n* = 2), and elite (i.e., competing at an international level, either against athletes of the same age group or for your country; *n* = 2). The main participants were primarily of White ethnicity (58.3%). One quarter (25.0%) of the participants were West Asian, and the remaining participants were either Arab, Black, Indigenous, or South East Asian.

#### Phase II Procedures

Upon arrival to the testing area, all participants were asked to complete several questionnaires (see below). Following a warm-up (i.e., jogging and dynamic stretches), all participants were asked to watch a short video that explained the feedback they would receive after each sprint. Kinematic data were collected using inertial sensors worn on the participant's trunk, and right and left ankles (see Phase II Instruments section). After preparation, each participant performed four sets of 40 meter sprint tests on a straight path in an indoor varsity-standard running track. Immediately after each sprint, participants received a combination of KR (total time) and KP (stride length, stride frequency, side-to-side sway, and front-to-back sway) biomechanical feedback that compared their individual performance to the gold standard data. The time of the first sprint test was used to establish participant baseline sprint performance. The time of the fourth sprint test established the final performance record for each participant. Three minutes of rest were provided between trials to avoid fatigue. Participants were permitted more rest if requested, but none asked for extra time.

#### Phase II Instruments

##### Demographics

Participants reported their age, sex, ethnicity, medical history, as well as their sports participation and training history within the past 12 months (Daniels and Leaper, 2006; Mosewich, 2008).

##### Self-Compassion Scale

An athlete-specific version of the Self-Compassion Scale (SCS-AV: Killham et al., 2018) was used to measure participants' self-compassion in sport. The original 26-item Self-Compassion Scale (SCS) developed by Neff (Neff, 2003b) is characterized by six subscales: Self-Kindness (five items; e.g., “I'm kind to myself when I'm experiencing suffering”), Self-Judgment (five items; e.g., “When times are really difficult, I tend to be tough on myself”), Common Humanity (four items; e.g., “When things are going badly for me, I see the difficulties as part of life that everyone goes through”), Isolation (four items; e.g., “When I fail at something that's important to me I tend to feel alone in my failure”), Mindfulness (four items; e.g., “When something painful happens I try to take a balanced view of the situation”), and Over-Identification (four items; e.g., “When I'm feeling down I tend to obsess and fixate on everything that's wrong”). The SCS-AV has been tailored for athletes by incorporating language specific to a sport context (e.g., substituting “athletes” for “people”), rather than measuring self-compassion at a domain-general level. Items from the self-judgment, isolation, and over-identification subscales are all phrased negatively, and were reverse scored before computing the total scale mean. Participants responded to items on a 5-point scale (1 = almost never, 5 = almost always), with higher composite scores reflecting higher levels of sport-specific self-compassion. Validity and reliability evidence supporting the use of the SCS-AV has been reported in the sport literature (α = 0.85–0.88: Killham et al., 2018).

##### Self-Esteem Scale

The Rosenberg Self-Esteem Scale (RSES: Neff, 2003) was used to assess athlete self-esteem (α = 0.82–0.87: Rosenberg, 1965). The RSES is a unidimensional questionnaire made up of 10 items (e.g., “I feel that I am a person of worth, at least on an equal plane with others”). Some items are reverse coded. Respondents were asked to rate items on a four-point scale (0 = strongly disagree, 3 = strongly agree), with higher total scores indicating higher levels of sport self-esteem.

##### Concern Over Mistakes Scale

The Concern Over Mistakes subscale of the Sports Multidimensional Perfectionism Scale-2 (Sport-MPS-2) was used to assess athletes' sport-specific concern over mistakes (α = 0.79: Li et al., 2018). The subscale consists of eight items (e.g., “The fewer mistakes I make in competition, the more people will like me”). Participants responded to items on a 5-point scale (1 = strongly disagree, 5 = strongly agree), with higher total scores reflecting higher levels of sport-specific concern over mistakes.

##### Self-Criticism Scale

To measure participants' levels of self-criticism, the state self-criticism measure by Gilbert and Procter (2006) was used (α = 0.84: Gotwals and Dunn, 2009). This seven-item scale asks participants about the frequency, power, intrusiveness, length, and the difficulty of distraction from their self-critical thoughts (e.g., “How long did your self-critical thoughts last?”). In this study, athletes were asked to complete a self-criticism questionnaire both pre- and post-sprint trials. To better suit the study purpose, the wording of the questionnaires was altered. Pre-sprint trials, participants were asked to think about the past year and rate their critical thoughts. Post-sprint trials, participants were asked to consider the biomechanical feedback given about their sprinting performance and rate the severity of their self-critical thoughts related to receiving and implementing this feedback. Participants responded on a ten-point scale (1 = not at all, 10 = a lot of the time), with higher total scores indicating higher levels of self-criticism.

##### Performed Scenario Scale

To assess their overall experiences after completing the sprint task and receiving biomechanical feedback, athletes were asked to rate their emotions, thoughts, and reactions using scales adapted from Leary et al. (2007) (see Supplementary Data for further information). To capture athlete emotions, four unique dimensions were assessed: Sadness (i.e., sad, dejected, down, and depressed), Anxiety (i.e., nervous, tense, worried, and anxious), Anger (i.e., angry, irritated, mad, and hostile), and Embarrassment (i.e., embarrassed, humiliated, disgraced, and ashamed). Each dimension was made up of four items, and participants rated each item based on a six-point scale (1 = not at all, 6 = extremely). A total scale score was calculated, with higher scores indicating higher levels of total negative affect.

To assess athlete thoughts after receiving biomechanical feedback, four unique dimensions were assessed: Catastrophizing (one item; e.g., “This is awful!”), Personalizing (three items; e.g., “I am such a loser”), Equanimity (two items; e.g., “In the long run, this doesn't really matter”), and Humor (one item; e.g., “This is sort of funny”). Participants rated each item based on a five-point scale (1 = I did not think this thought at all, 5 = I kept thinking this thought). Total scores were calculated for each dimension, with higher scores indicating higher levels of that type of thought. A second set of questions was asked to further assess athlete emotions. There were six items in total: (a) I seem to have bigger problems than most people do; (b) I'm a loser; (c) This isn't any worse than what lots of other people go through; (d) Why do these things always happen to me?; (e) In comparison to other people, my life is really screwed up; and (f) Everyone has a bad day now and then. This second set of questions was rated on a six-point scale (1 = not at all, 6 = extremely), and the score of each individual item was used in further analysis.

To evaluate athlete reactions, participants were asked to complete two sets of questions. First, they were asked to rate the degree to which they displayed the following reactions after receiving biomechanical feedback on their sprinting performance: (a) Remained relatively calm and unflustered; (b) Overreacted; (c) Experienced strong emotions but did not get carried away with them; (d) Had no emotional reaction whatsoever; (e) Took the feedback in stride; (f) Set aside the feedback quickly in order to deal with my emotions; and (g) Replayed the feedback in my mind for a long time afterward. Participants responded on a six-point scale (1 = not at all, 6 = extremely). Some items were reverse scored (i.e., [b] and [g]). Higher total scores reflected higher levels of behavioral equanimity. The second set of questions asked about the degree to which each athlete reacted after receiving biomechanical feedback: (a) I tried to be kind to myself; (b) I tried to make myself feel better; (c) I was really hard on myself; (d) I kept the feedback in perspective; (e) I tried to do things to take my mind off of the feedback; (f) I expressed my emotions to let off steam; (g) I took steps to fix the problem or made plans to do so; (h) I sought out the company of others; and (i) I gave myself time to come to terms with it. Participants responded on a six-point scale (1 = not at all, 6 = extremely) and the score of each individual item was used in further analysis.

##### Timing and Inertial Sensors

The first author led all data collection. A set of electronic timing gates (Freelap Pro Coach BLE, 0.02 second accuracy, Freelap, USA) were used to time the sprinters' performance over the 40 meters distance. The same timing gates were used for both the gold standard athletes and the main participants. These timing gates used a chip that attached to the athlete's waistband. The time data transferred from the chip to a transmitter placed at the end of the track. As the athlete passed the transmitter, the time data was sent to a smart phone via Bluetooth connection and then manually recorded. The remaining biomechanical feedback data were collected by small lightweight wearable wireless inertial sensors (Opal, APDM Inc., Portland, OR; 43.7 mm × 39.7 mm × 13.7 mm; <25 g). The inertial sensors contain three-dimensional accelerometers, gyroscopes, and magnetometers that provide kinematic information about sensor orientation, acceleration, and angular velocity. Data were collected at a sample rate of 128 Hz and transmitted wirelessly to a PC-based laptop. The participants wore three inertial sensors: one each on the trunk, right ankle (RA), and left ankle (LA). The sensors on the ankles were securely fixed by loop and hook fabric straps on the anterior surface of the shin near the ankle to prevent motion artifact noise. These straps did not limit the range of motion of the ankle joint. The trunk sensor was fixed over the sternum using custom light-weight straps.

The angular velocity of the RA and LA extracted from sensors was analyzed using a custom algorithm written in MATLAB (R2019a, Mathworks, Natick, MA) to calculate the total number of steps for each trial. The sagittal angular velocity of the RA and LA sensors were first filtered using a fourth order low pass Butterworth filter with a cut off frequency of 5 Hz. Distinct angular velocity peaks associated with each step were then identified using a thresholding technique, which gave a count of the number of steps in the data. The step indices were used in conjunction with the trunk resultant acceleration to automatically identify the first step. The externally measured sprint time was then used to determine the number of steps that were part of the 40-meter sprint. Step time was calculated by dividing the step count by the sprint time. The step frequency was calculated as the inverse of step time and multiplied by 60 to express it in units of steps per minute (i.e., $Step\;frequency\;\left[\frac{step}{minute}\right]=\frac{1}{Step\;time\;\left(seconds\right)}\;\times \;60$). The average step length was calculated by dividing the 40-meter distance by the number of steps. To normalize the step length, values were divided by each participant's height. Data from the trunk sensor was used to calculate the side-to-side and front-to-back sway of the trunk using another custom MATLAB routine. Raw trunk angular velocity data in each movement axis was partitioned using the step timing information obtained from the ankle sensors. The angular velocity data for each step was then numerically integrated to obtain the angular displacement (i.e., sway) for each step. The range of motion of both side-to-side sway and front-to-back sway were calculated.

Feedback was given on a laptop screen using a custom MATLAB routine. After each sprint trial, athletes received their step length and frequency data in the form of a two-row bar graph. The top bar represented sex-matched gold standard data and the lower bar was the participant's performance for that trial (Figure 1). Numerical data for these variables were also presented beside the bar graphs. In addition, explicit text was provided for the participant to read about how their data differed from the gold standard. This feedback was either positive, meaning that the participant had exceeded the gold standard metric, or negative, meaning that the participant's results were worse than the gold standard metric. For example, participants would be told that their sprint time was SLOWER or FASTER than the gold standard. For feedback about side-to-side and front-to-back sway of the trunk, visual feedback in the form of two arcs showing the range of angular movement (with the gold standard data above the participant data) was given. To examine if there was any significant change in sprint performance after receiving and implementing feedback, all the data for each trial was saved for further analysis.

#### Phase II Data Analysis

Statistical analysis was computed using SPSS v22 (IBM Corp., Armonk, NY, USA) with the alpha level set at 0.05. Data were screened for missing responses and normality. No missing data were identified across any of the scales and subscales; thus, data from 48 participants were used for hypothesis testing^1^. Self-compassion, self-esteem, concern over mistakes, and pre- and post-trials self-criticism scales were normally distributed, whereas most of the items in the post-trials emotions, thoughts, and reactions subscales violated the normality assumption and were positively skewed. Based on the protocol used by other self-compassion studies in sport (Mosewich et al., 2011; Reis et al., 2015), which described that their substantive conclusions did not change after transforming similar data, we used the non-transformed scales values in all analyses.

A one-tailed Pearson correlation analysis was performed to test if the relationships among self-compassion, self-esteem, concern over mistakes, and pre-trials self-criticism were in the same directions as predicted in Hypothesis 1. To test Hypothesis 2, a one-tailed Pearson correlation analysis was used to determine the correlations between self-compassion, self-esteem, concern over mistakes, and pre-trials self-criticism with post-trials self-criticism, emotions, thoughts, and reactions. Hypothesis 3 was tested using a within-between 4 (sprint trial) × 2 (sex) repeated measures ANOVA to examine if any significant changes occurred in participant biomechanical variables after receiving and implementing biomechanical feedback. Afterwards, a moderated regression analysis was conducted using self-compassion as a moderator between the first and fourth sprint times. The fourth sprint time was entered as a dependent variable. The first sprint time was entered into the model in step 1. Self-compassion scores were introduced in step 2. Finally, to measure whether self-compassion was a moderator for change in sprint performance, the interaction between first sprint time and self-compassion (i.e., first sprint time × self-compassion) was introduced in step 3.

## Results

Descriptive statistics and the internal reliabilities for all psychological scales and subscales are presented in Table 2.

**Table 2:** Descriptive statistics and internal reliabilities of psychological scales and subscales for Phase II participants.

**Variable**	***M***	**SD**	**α**
Self-compassion	3.47	0.55	0.86
Self-esteem	3.20	0.40	0.79
Concern over mistakes	2.58	0.87	0.87
Pre-trials self-criticism	4.35	1.42	0.76
Post-trials self-criticism	3.62	1.25	0.71
Emotions (total negative affect)	20.73	7.78	0.86
**Thoughts (set one)**			
Catastrophizing	2.58	1.23	0.55
Personalizing	2.79	1.15	–
Equanimity	1.56	1.03	0.30
Humorous	1.73	1.16	–
**Thoughts (set two)**			
I seem to have bigger problems than most people do	1.27	0.54	–
I'm a loser	1.04	0.20	–
This isn't any worse than what lots of other people go through	1.87	1.23	–
Why do these things always happen to me?	1.02	0.14	–
In comparison to other people, my life is really screwed up	1.15	0.41	–
Everyone has a bad day now and then	1.73	1.16	–
**Reactions (set one)**			
Behavioral equanimity	28.73	3.51	0.86
**Reactions (set two)**			
I tried to be kind to myself	3.75	1.30	–
I tried to make myself feel better	3.45	1.54	–
I was really hard on myself	2.23	1.30	–
I kept the feedback in perspective	4.52	1.13	–
I tried to do things to take my mind off of the feedback	1.50	0.92	–
I expressed my emotions to let off steam	1.46	0.99	–
I took steps to fix the problem or made plans to do so	4.06	1.69	–
I sought out the company of others	1.73	1.16	–
I gave myself time to come to terms with it	2.44	1.44	–

*α refers to Cronbach's alpha measure of internal consistency. For single items (i.e., personalizing and humorous thoughts, thoughts [set two] and reactions [set two]), internal consistency values could not be calculated. The ranges of the measures included in the above table are as follows: self-compassion (range = 1–5); self-esteem and concern over mistakes (range = 1–4); pre- and post-trials self-criticism (range = 1–10); total negative affect (range = 16–96); catastrophizing and personalizing thoughts (range = 2–10); equanimity and humorous thoughts (range = 1–5); set two thoughts (range = 1–6); behavioural equanimity (range = 7–42); set two reactions (range = 1–6)*.

### Hypotheses Testing

#### Hypothesis 1: Relationships Between Psychological Variables at Baseline

Hypothesis 1 was predicated on the relationships between psychological scales prior to the sprint tests and was supported by our results (see Table 3). We found negative correlations between self-compassion, and concern over mistakes and pre-trials self-criticism. Furthermore, we found a positive correlation between self-compassion and self-esteem.

**Table 3:** One-tailed Pearson correlations before receiving feedback for Phase II participants.

**Variable**	**Self-esteem**	**Concern over mistakes**	**Pre-trials self-criticism**
Self-compassion	0.57**	−0.69**	−0.52**
Self-esteem	–	−0.46*	−0.50**
Concern over mistakes		–	0.50**

*p <0.05; **p <0.01.

#### Hypothesis 2: Relationships Between Psychological Variables Before and After Sprint Testing

The correlations among all variables relevant to Hypothesis 2 are shown in Table 4. Supporting Hypothesis 2a, there was a negative correlation between post-trials self-criticism and both self-compassion and self-esteem. Hypothesis 2b was partially supported, as there was a negative correlation between both self-compassion and self-esteem with maladaptive emotions (i.e., total negative affect) and catastrophizing thoughts, and a positive correlation with some positive reactions (i.e., “I tried to be kind to myself,” and “I tried to make myself feel better”). However, Hypothesis 2b was not fully supported as no relationship was found between both self-compassion and self-esteem and the remaining thoughts and reactions. Hypothesis 2c was fully supported as there was a positive correlation between post-trials self-criticism and both concern over mistakes and pre-trials self-criticism. Lastly, partial support was found for Hypothesis 2d as both concern over mistakes and post-trials self-criticism were positively correlated to maladaptive emotions (i.e., total negative affect), but were unrelated to any other thoughts or reactions.

**Table 4:** One-tailed Pearson correlations of psychological scales and subscales for Phase II participants.

**Variable**	**Self-compassion**	**Self-esteem**	**Concern over mistakes**	**Pre-trials self-criticism**
Post-trials self-criticism	−0.38**	−0.36**	0.25*	0.59**
Total negative affect	−0.38**	−0.55**	0.32*	0.31*
**Thoughts (set one)**				
Catastrophizing	−0.37**	−0.35**	0.27*	0.02
Personalizing	−0.34**	−0.18	0.13	0.17
Equanimity	−0.24*	−0.11	0.08	0.06
Humorous	−0.03	0.02	−0.02	−0.11
**Thoughts (set two)**				
I seem to have bigger problems than most people do	−0.11	−0.24	0.18	0.20
I'm a loser	−0.19	−0.34**	0.04	0.06
This isn't any worse than what lots of other people go through	0.03	0.07	−0.10	−0.18
Why do these things always happen to me?	−0.04	−0.00	−0.10	0.10
In comparison to other people, my life is really screwed up	−0.06	−0.31*	0.10	−0.00
Everyone has a bad day now and then	−0.11	−0.21	0.10	−0.10
**Reactions (set one)**				
Behavioral equanimity	0.22	0.32*	−0.08	−0.15
**Reactions (set two)**				
I tried to be kind to myself	0.38**	0.31*	−0.33*	−0.22
I tried to make myself feel better	0.33*	0.32*	−0.23	−0.18
I was really hard on myself	−0.18	−0.24*	0.21	0.23
I kept the feedback in perspective	0.27*	−0.06	−0.15	−0.01
I tried to do things to take my mind off of the feedback	0.10	−0.04	−0.09	−0.02
I expressed my emotions to let off steam	−0.02	0.21	−0.00	−0.07
I took steps to fix the problem or made plans to do so	0.23	0.32*	0.10	0.05
I sought out the company of others	0.05	−0.02	0.03	0.10
I gave myself time to come to terms with it	0.23	0.19	−0.09	0.02

*p <0.05; **p <0.01.

#### Hypothesis 3: Changes Between Biomechanical Variables Before and After Sprint Testing

**Figure 2:**
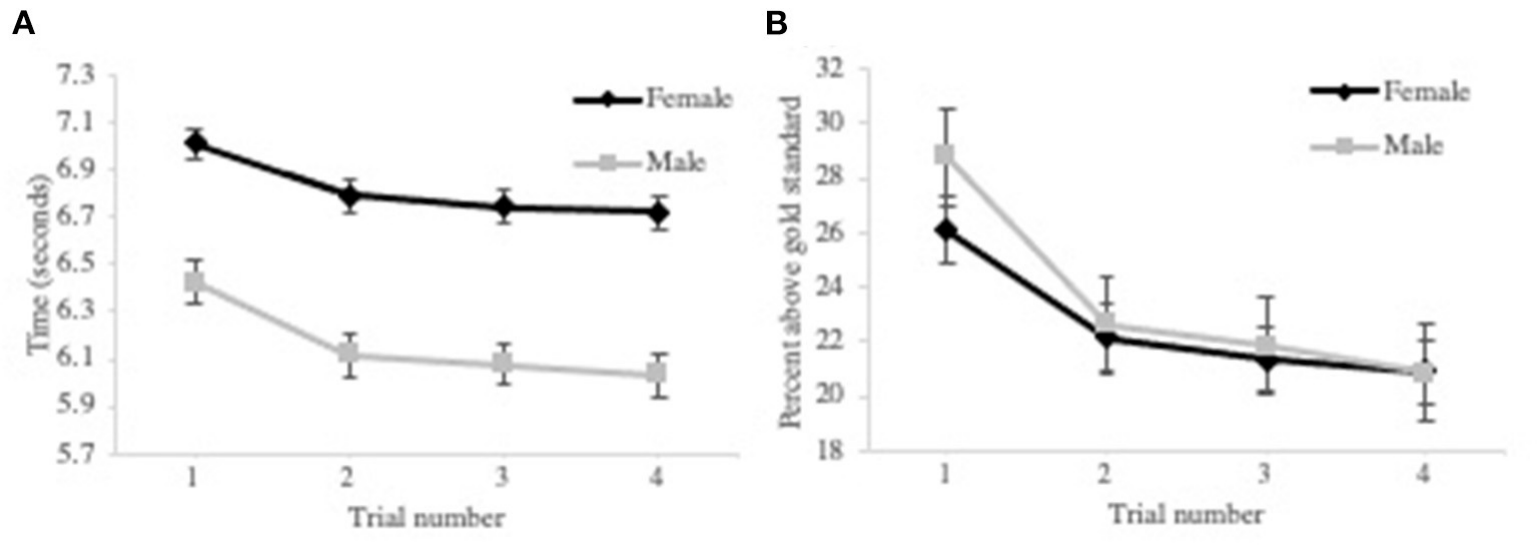
See effect on Phase II participants sprint time. Graph **(A)** represents the original sex-specific mean for each sprint time trial. Graph **(B)** represents the sex-specific sprint time mean expressed as the difference between the subject's sprint time and the gold standard as a percent of the gold standard obtained from the participants for each sprint time trial.

We predicted that athletes with higher levels of self-compassion would have significantly better sprint performances after receiving and implementing biomechanical feedback. Descriptive statistics for the biomechanical variables are represented in Table 5. The results of a within-between repeated measures ANOVA revealed that there was a change in sprint time [*F*_(1.67, 76.78)_ = 21.61, *p* < 0.001], step length [*F*_(2.43, 111.79)_ = 22.72, *p* < 0.001], side-to-side sway [*F*_(2.40, 110.56)_ = 18.95, *p* < 0.001], and front-to-back sway [*F*_(2.73, 125.64)_ = 8.10, *p* < 0.001] over sprint trials. The pairwise comparison of the means with Bonferroni adjustment (α = 0.05/6) showed that the changes in all four variables occurred between the first sprint set and the second, third, and fourth sprint sets. There were no interactions between these biomechanical variables and sex, but there were sex effects on sprint time [*F*_(1, 46)_ = 22.94, *p* < 0.001], and front-to-back sway [*F*_(1, 46)_ = 13.362, *p* < 0.05]. To account for this, sprint time and sway were expressed as percentages of the respective sex-based gold standards and the analysis was re-run. All other results remained the same, but the sex effects were no longer significant (see Figure 2).

**Table 5:** Pairwise means comparison of biomechanical variables between sprint trials of Phase II participants.

**Variable**	**Mean difference**	**SE**	***P* ^a^**
**Sprint time**
1st−2nd	0.26*	0.05	0.000
1st−3rd	0.31*	0.06	0.000
1st−4th	0.34*	0.07	0.000
**Step length**
1st−2nd	−0.05*	0.01	0.000
1st−3rd	−0.06*	0.01	0.000
1st−4th	−0.50*	0.01	0.000
**Step frequency**
1st−2nd	1.90	1.03	0.428
1st−3rd	1.49	0.88	0.575
1st−4th	0.22	1.12	1.000
**Front-to-back sway**
1st−2nd	−1.37*	0.40	0.008
1st−3rd	−1.52*	0.39	0.002
1st−4th	−1.29*	0.38	0.008
**Side-to-side sway**
1st−2nd	−1.02*	0.23	0.000
1st−3rd	−1.66*	0.31	0.000
1st−4th	−1.89*	0.32	0.000

*The mean difference column represents the differences between the means of each biomechanical variable at each given sprint trial. SE, standard error; P^a^, Significance level with Bonferroni adjustment*.

*p <0.05/6.

As the changes in sprint time, step length, side-to-side sway, and front-to-back sway were similar after receiving biomechanical feedback, we chose to use sprint time as the main indicator of sprint performance to test Hypothesis 3 as it is likely the most tangible and familiar sprint-related concept to the reader and layman alike. A one-tailed Pearson correlation was used to assess the relationships between sprint time and psychological factors measured pre- and post-sprint trials. There were no significant relationships between sprint time and self-compassion, self-esteem, concern over mistakes, and post-trials self-criticism. The correlations between sprint time and post-trials self-criticism, emotions, thoughts, and reactions are presented in Table 6.

**Table 6:** One-tailed Pearson correlations after receiving feedback after each sprint trial for Phase II participants.

**Variable**	**1st sprint trial**	**2nd sprint trial**	**3rd sprint trial**	**4th sprint trial**	**4th−1st sprint trial**
Post-trials self-criticism	0.08	0.21	0.16	0.19	0.10
Total negative affect	0.33*	0.28**	0.33*	0.31*	−0.17
**Thoughts (set one)**
Catastrophizing	−0.01	0.02	0.01	0.10	0.13
Personalizing	0.23	0.14	0.07	0.07	−0.30*
Equanimity	−0.03	−0.07	0.03	0.02	0.082
Humorous	−0.37**	0.33*	0.25*	0.21	−0.35**
**Thoughts (set two)**
I seem to have bigger problems than most people do	0.19	0.28*	0.34**	0.37**	0.14
I'm a loser	−0.04	−0.01	−0.01	0.07	0.15
This isn't any worse than what lots of other people go through	0.11	0.09	0.10	0.07	−0.10
Why do these things always happen to me?	0.00	0.07	0.07	0.09	0.11
In comparison to other people, my life is really screwed up	0.18	0.16	0.20	0.16	−0.11
Everyone has a bad day now and then	0.17	0.10	0.13	0.21	−0.03
**Reactions (set one)**
Behavioral equanimity	−0.07	0.01	−0.06	−0.04	0.07
**Reactions (set two)**
I tried to be kind to myself	0.10	0.05	0.02	0.02	−0.14
I tried to make myself feel better	0.26*	0.19	0.13	0.12	−0.28*
I was really hard on myself	0.20	0.21	0.18	0.21	−0.08
I kept the feedback in perspective	0.03	0.02	0.01	−0.09	−0.16
I tried to do things to take my mind off of the feedback	0.34**	0.20	0.23	0.15	−0.38**
I expressed my emotions to let off steam	0.18	0.08	0.08	0.05	−0.23
I took steps to fix the problem or made plans to do so	0.14	0.13	0.03	−0.06	−0.30*
I sought out the company of others	0.27*	0.07	0.07	0.02	−0.42**
I gave myself time to come to terms with it	0.36**	0.25*	0.22	0.11	−0.45**

*p <0.05; **p <0.01.

Further analyses were completed to evaluate the relationship between self-compassion and the sprint performances of athletes after receiving and implementing biomechanical feedback. A moderated regression analysis was run with the interaction between first sprint time and self-compassion entered as a moderator between the first and fourth sprint times. This interaction did not predict any unique variance in sprint performance, thus self-compassion was not a moderator for change in sprint performance (*R*^2^ = 0.642, Δ*R*^2^ = 0.10, *p* > 0.05). In sum, the results of these analyses refuted Hypothesis 3. While sprint performance was improved after receiving biomechanical feedback, there was no relationship between the improvement of sprint performance and athlete self-compassion.

## Discussion

The purpose of this research was to explore whether self-compassion, self-esteem, concern over mistakes, and self-criticism could predict athletes' responses to biomechanical feedback after a series of sprint performances, and whether self-compassion could buffer against any maladaptive emotions, thoughts, and reactions experienced after receiving and implementing this feedback. We found that athletes with higher levels of self-compassion demonstrated higher levels of self-esteem, less concern over mistakes, and lower levels of pre-trials self-criticism. We also found that athletes with higher levels of self-compassion prior to sprint performance experienced less self-critical thoughts following biomechanical feedback and subsequent sprint trials. Though the results of this study provide some support for the effectiveness of self-compassion in promoting adaptive emotions, thoughts, and reactions in response to sprint performance biomechanical feedback, we found little evidence suggesting that high levels of self-compassion led to more effective implementation of the feedback and improved sprint performance. More specifically, self-compassion was not a moderator of change in sprint performance across trials, and the relationship between self-compassion and sprint performance was non-significant.

Our study supported the results of previous studies, in that we found negative correlations among self-compassion and concern over mistakes, pre-trials self-criticism, and post-trials self-criticism (Mosewich et al., 2013). We also found negative correlations between self-esteem and concern over mistakes, pre-trials self-criticism, and post-trials self-criticism (Reis et al., 2019). We also found that both pre-trials self-criticism and concern over mistakes were positively correlated with post-trials self-criticism and emotions (i.e., total negative affect), though the majority of the relationships between pre-trials self-criticism and concern over mistakes, and post-trials thoughts and reactions were non-significant. A possible reason for these non-significant relationships could have been our participants' high mean levels of self-compassion—descriptive analyses revealed that athletes who participated in this study had higher levels of self-compassion (*M* = 3.47, *SD* = 0.55) in comparison to Reis et al.'s (*M* = 3.10, *SD* = 0.58; 2015) and Leary et al.'s (Study 1: *M* = 3.15, *SD* = 0.63; Study 2: *M* = 3.03, *SD* = 0.58; Study 5: *M* = 3.08, *SD* = 0.58; 2007) participants. Self-compassion can be used as a coping strategy to reduce an individual's self-criticism and concern over mistakes (Gilbert and Procter, 2006; Mosewich et al., 2013). Additionally, self-compassion can reduce maladaptive emotions, thoughts, and reactions by fostering more positive perceptions of the self (Neff, 2003b). Perhaps the relatively high self-compassion scores of the athletes included in this study made it less likely that they would experience adverse reactions to the biomechanical feedback, and thus experienced less self-criticism and concern over mistakes. In other words, it seems possible that the athletes simply did not experience the biomechanical feedback as an emotionally difficult setback that fostered self-criticism and concern over mistakes, as they were already viewing the feedback from a more self-compassionate lens.

Previous studies have emphasized the positive role of self-compassion in coping with emotionally difficult sport-related experiences (e.g., being responsible for a team failure: Mosewich et al., 2013; Ferguson et al., 2014; Reis et al., 2015). Some of our results are in line with these findings. Specifically, the correlations between self-compassion and emotions (i.e., total negative affect), and some thoughts (i.e., catastrophizing, personalizing, and equanimity) and reactions (i.e., “I tried to be kind to myself,” “I tried to make myself feel better,” and “I kept the feedback in perspective”) were positive, thus emphasizing the potential positive effects of self-compassion on athletes' responses to challenging sport situations. Conversely, the relationships between self-compassion and the remaining measured thoughts and reactions (e.g., humorous thoughts, behavioral equanimity) were not significant and did not align with extant research. Perhaps athletes are so habituated to receiving performance-related feedback—though not necessarily biomechanical feedback—over their many years of training that they might already have a set of coping skills in place to cope with feedback. This explanation might also support why self-esteem was sufficient for predicting athlete responses to the given feedback. Receiving and implementing biomechanical feedback also may not be a situation in which self-compassion is essential, as it might be one of several effective coping skills that athletes have available to them.

We found that athletes receiving biomechanical feedback for the first time (i.e., after their first sprint set) significantly improved their following sprint performance (i.e., second sprint set). A wide range of studies provide compelling evidence that support the positive effect that biomechanical feedback has on performance enhancement (Broker et al., 1989; Sanderson and Cavanagh, 1990; Mendoza and Schöllhorn, 1993); however, no significant differences were observed in athlete performance between their second, third, and fourth sprint performances. One possible explanation could be the possible misinterpretation of biomechanical feedback by study participants. Preatoni et al. (2013) highlighted the importance of translating biomechanical feedback into easily understandable information for athletes. No prescriptive feedback (i.e., error correction) was provided by our study experimenter which may have limited impact of the biomechanical feedback and the ability of athletes to implement the feedback in subsequent performances.

Another possible explanation for the improvement in athlete performance between only the first and second sprint sets could be attributed to a practice effect. Athletes may have improved their sprint sets due to familiarization with test protocols and equipment (i.e., inertial sensors and timing gate chip) rather than feedback effects. To ascertain if familiarization was indeed responsible for this performance improvement, a secondary experiment was performed on 10 athletes that were not part of the current study. Results revealed that there was no significant effect on sprint performance between first and second trials^2^. The data from this secondary study suggest that the performance improvement seen between the first and second trials in the main study were unlikely to have been caused by practice effects.

In the current study, a combination of KR and KP biomechanical feedback was provided to athletes at the end of each trial. The athletes received KR feedback on sprint time and KP feedback on their step frequency, step length, and trunk sway. As discussed above, we found a significant improvement between the first and second trial sprint times (i.e., KR feedback), but no significant changes were observed in subsequent trials. One possible reason for this lack of continued change could be due to the frequency of providing KR feedback to our participants. Previous studies have shown that less frequent KR feedback (i.e., having some no-feedback trials) can improve the motor skill acquisition more efficiently than when more frequent KR feedback is given (Salmoni et al., 1984). It has been posited that while frequent KR feedback might guide a learner to target behavior, it may later prevent individuals from using intrinsic feedback processing and error detection and might cause learner dependency (Salmoni et al., 1984). Additionally, we found that the effects of KP feedback were also not consistent across each of the measured variables. In the extant literature, evidence suggests that frequently delivered KP feedback is a superior guide during basic skill acquisition, particularly for novices (Wulf et al., 1994; Wulf and Shea, 2002). Frequent KP feedback has also been shown to significantly improve complex movements (i.e., sports-specific skills requiring whole-body movements with many degrees of freedom: Wulf et al., 1994). More specifically, when Wulf et al. (1998) provided KP feedback about force onset to participants participating in ski simulator training, those who received KP feedback at 100% frequency demonstrated superior performance improvement over those who only received KP feedback at 50% frequency. Perhaps providing our main study participants with higher frequency KP feedback may have led to improvements in step frequency, step length, and trunk sway performances over sprint trials. It is also possible that the main study participants may not have known how to implement new techniques to efficiently improve all of the examined metrics of sprint performance, as receiving this specific biomechanical feedback was likely new to them. For example, participants who had less side-to-side trunk sway than the gold standard may have known that they *should* increase their trunk sway to improve their sprint time, but they may not know *how* to do so. Providing additional prescriptive feedback may have facilitated participants' interpretations of biomechanical feedback received about their sprint performance and may have led to continued improvements in these kinematic variables over sprint trials.

### Limitations

A limitation of our study was the ability to fully control the testing environment. Sports performance can vary based on the presence of others (Geisler and Leith, 1997). All the data from the main participants were collected on a public indoor running track; however, to help control for potential distraction of other people, testing hours were chosen to minimize the effects of external factors (e.g., number of non-participants present at the location, audience feedback, noise, etc.), and only study participants were allowed to enter the test site. Having said this, despite our best attempts, the testing environment did vary to some degree across participants.

Another limitation of this research may have been the limited variability of our participants' baseline levels of self-compassion. One of the primary objectives of our study was to explore whether self-compassionate athletes had better sprint performance than their less self-compassionate peers after receiving feedback. Purposefully recruiting athletes with varying levels of self-compassion may help in examining the responses to biomechanical feedback in athletes as this would have likely resulted in a greater variance in the other psychological variables we measured and, consequently, the effectiveness of using self-compassion as a coping strategy.

A final limitation involved our inability to stratify our sample based on sex due to the size of our sample pool, which impeded our ability to draw any conclusions about the presence of sex-based differences in the measured psychological variables. Some evidence exists demonstrating that males (and men) and females (and women) score differently on measures of self-compassion (Lizmore et al., 2017), self-esteem (Li et al., 2018), concern over mistakes (Cremades et al., 2013), and self-criticism (Luyten et al., 2007), but other researchers (Gotwals et al., 2003; Anshel et al., 2009; Huysmans and Clement, 2017; Dunn et al., 2021) have found the opposite to be true (i.e., no significant differences between sexes or gender, see footnote 1). Future researchers should consider stratifying their sample by sex, in addition to controlling for possible differences in gender in their analyses.

### Future Directions

There are several considerations that can be implemented to develop more effective biomechanical feedback for athletes. The participants in our study were not track and field athletes and it is likely that some of the biomechanical content of the feedback may have been new to them. As such, a combination of coach/expert feedback and tutorial videos might have provided better guidance for athletes (Preatoni et al., 2013) and subsequently improved their performance. Future studies may find different results if both descriptive and prescriptive biomechanical feedback are delivered to study participants. Second, the emotional reactions of individuals during sport performance can change across time points, especially in stressful situations (Crocker et al., 1998; Cerin et al., 2000). Measuring discrete emotions that might be relevant to the experience of receiving biomechanical feedback (including both negative and positive emotions; e.g., pride, joy, and happiness) might garner more information about the emotional experiences of athletes receiving biomechanical feedback about their performance. Next, although the validity and reliability of composite self-compassion scores has been well-documented (Neff, 2003b), analyzing the self-compassion subscales offers an alternative approach to explore the associations among self-compassion and adaptive emotions, thoughts, and reactions in response to biomechanical feedback. Previous studies show the utility of specific subscales—such as Common Humanity and Mindfulness—in decreasing self-criticism (Jopling, 2000) and openness to feedback (Neff, 2003a). It seems reasonable that some self-compassion subscales would be more strongly related to athletes' responses to feedback than a total score. Lastly, further research studying the effects of self-compassion inductions on sport performance and athletes' responses to biomechanical feedback is needed. Practicing self-compassion could increase athletes' awareness of their emotions, thoughts, and reactions (Leary et al., 2007; Reis et al., 2015), and—as a result of being more self-compassionate—might experience more adaptive responses to biomechanical feedback.

## Conclusion

Our results suggest that self-compassion can play a nuanced role in athlete coping and sport performance. Self-compassion was related to adaptive psychological characteristics prior to sprint trials (i.e., positively correlated with self-esteem, negatively related to self-criticism, and concern over mistakes) and seemed to attenuate athletes' negative responses to biomechanical feedback after sprint performance (e.g., negative affect, negative thoughts, and reactions). However, higher levels of self-compassion did not seem to help athletes perform better after receiving biomechanical feedback. We also found that the specific type and frequency of the objective biomechanical feedback used in this study did not appear to improve sprinting performance in this population. While the first presentation of biomechanical feedback significantly improved the sprint time, step length, side-to-side sway, and front-to-back sway in participants, these enhancements were not continually improved across subsequent attempts. There is a possibility that providing more guidance (i.e., prescriptive coach feedback) might have increased the impact of biomechanical feedback on athlete performance. To further advance the literature, sport researchers might consider different approaches to providing biomechanical feedback to their participants, measuring additional emotions that might be relevant to the experience of receiving feedback, approaching the measurement of self-compassion from a sub-scale perspective, and implementing self-compassion inductions prior to performance trials. We believe that further efforts made to identify the constellation of psychological skills that can lead to the efficient execution of feedback in competitive environments will ultimately enhance sport performance and increase adaptive psychological outcomes for athletes.

## Data Availability Statement

The datasets presented in this article are not readily available due to lack of participant consent. Requests to access group-only data should be directed to joel.lanovaz@usask.ca.

## Ethics Statement

The studies involving human participants were reviewed and approved by the University of Saskatchewan Research Ethics Board. Written informed consent to participate in this study was provided by the participants.

## Author Contributions

YAA was supervised by JLL and KCK. YAA, KCK, ARO, LJF, and JLL contributed to conception and design of the study. YAA collected all data and performed the statistical analysis. DLC had significant writing responsibilities for the manuscript version of the research. KCK was responsible for the acquisition of the financial support leading to this publication. All authors read, revised, and approved the submitted version of this manuscript.

## Funding

This research draws on research supported by the Social Sciences and Humanities Research Council of Canada (SSHRC), grant number: 862-2016-0004.

## Conflict of Interest

The authors declare that the research was conducted in the absence of any commercial or financial relationships that could be construed as a potential conflict of interest.

## Publisher's Note

All claims expressed in this article are solely those of the authors and do not necessarily represent those of their affiliated organizations, or those of the publisher, the editors and the reviewers. Any product that may be evaluated in this article, or claim that may be made by its manufacturer, is not guaranteed or endorsed by the publisher.

## Footnotes

^1^It was our intention to combine the psychological data from male and female subjects into a single sample, as assessing sex-based differences was not the primary purpose of this study. Preliminary independent *t*-tests were conducted to determine if sex-based differences existed across the four major psychological variables measured (female = 1, male = 2). No significant differences were found between males and females in each of the four psychological variables, including self-compassion [*t*_(1, 46)_ = 1.08, *p* = 0.29], self-esteem [*t*_(1, 46)_ = −1.42, *p* = 0.16], concern over mistakes [*t*_(1, 46)_ = 0.34, *p* = 0.74], pre-trial self-criticism [*t*_(1, 46)_ = 1.22, *p* = 0.23] and post-trial self-criticism [*t*_(1, 46)_ = 1.22, *p* = 0.23]. However, since the present study included a relatively small sample size, results related to the testing of sex differences should be interpreted with caution.

^2^Ten athletes (9 male: *M*_age_ = 21.8, *SD* = 3.1) performed four sets of 40-meter sprints with identical biomechanical data collection protocol as Phase II but with no psychological questionnaires. Participants received similar biomechanical feedback after sprinting as the original study, but only began to receive feedback after the second sprint trial (rather than the first). The result of a repeated measure ANOVA analysis revealed no significant sprint performance improvements between the first and second trials.
