# Supplementary material for: The Associations Among Self-Compassion, Self-Esteem, Self-Criticism, and Concern Over Mistakes in Response to Biomechanical Feedback in Athletes
Source: Front Sports Act Living. 2022 Apr 19;4:868576. doi: 10.3389/fspor.2022.868576 (PMC9062879; doi:10.3389/fspor.2022.868576)
Supplement: Supplementary file 1 [file Table_1.pdf]

Supplemental Data 1

The Reactions and Thoughts Scales Presented by Leary et al. (2007) and the Modified Scales Used for Performed Scenario

Reactions

**Hypothetical Scenario, Leary et al. (2007)**	**Question 1, Performed Scenario**
1. Remain *relatively* calm and unflustered	1. Remain calm and unflustered
2. Overreact	2. Overreact
3. Experience strong emotions but not get carried away *with them*	3. Experience strong emotions but not get carried away
4. Have no emotional reaction whatsoever	4. Have no emotional reaction whatsoever
5. Take the *situation* in stride	5. Take the *feedback* in stride
6. *Leave* the *situation* quickly in order to deal with my emotions	6. *Set aside* the *feedback* quickly in order to deal with my emotions
7. Replay the *situation* in my mind *for a long time afterwards*	7. Replay the *feedback* in my mind constantly

**Recalled Scenario, Leary et al. (2007)**	**Question 2, Performed Scenario**
1. I tried to be kind to myself	1. I tried to be kind to myself
2. I tried to make myself feel better	2. I tried to make myself feel better
3. I was really hard on myself	3. I was really hard on myself
4. I kept the *situation* in perspective	4. I kept the *feedback* in perspective
5. *I wanted to spend time alone*	5. *I tried to do things to take my mind off of the feedback*
6. I expressed my emotions to let off steam	6. I expressed my emotions to let off steam
7. I took steps to fix the problem *in a positive way* or *I* made plans to do so	7. I took steps to fix the problem or made plans to do so
8. I sought out the company of others	8. I sought out the company of others
9. I gave myself time to come to terms with it	9. I gave myself time to come to terms with it
10. *I tried to understand my emotions*	

Thoughts

**Hypothetical Scenario, Leary et al. (2007)**	**Question 1, Performed Scenario**
1. This is awful!	1. This is awful!
2. Everybody goofs up now and then	2. Everybody goofs up now and then
3. In the long run, this really doesn't matter	3. In the long run, this really doesn't matter
4. I am such a loser	4. I am such a loser
5. *I wish I could die*	5. This is embarrassing
6. This is sort of funny	6. This is sort of funny
7. I should have expected this would happen	7. I should have expected this would happen

**Recalled Scenario, Leary et al. (2007)**	**Question 2, Performed Scenario**
1. I seem to have bigger problems than most people do	1. I seem to have bigger problems than most people do
2. I'm a loser	2. I'm a loser
3. This isn't any worse than what lots of other people go through	3. This isn't any worse than what lots of other people go through
4. Why do these things always happen to me?	4. Why do these things always happen to me?
5. In comparison to other people, my life is really screwed up	5. In comparison to other people, my life is really screwed up
6. *I've had a really bad day-I need to do something nice for myself*	6. *Everyone has a bad day now and then*
